# Immunomodulatory effects of tumor Lactate Dehydrogenase C (LDHC) in breast cancer

**DOI:** 10.1186/s12964-025-02139-6

**Published:** 2025-03-19

**Authors:** Adviti Naik, Remy Thomas, Aljazi Al-Khalifa, Hanan Qasem, Julie Decock

**Affiliations:** 1 Translational Oncology Research Center, Qatar Biomedical Research Institute (QBRI), Hamad Bin Khalifa University (HBKU), Qatar Foundation (QF), Doha, Qatar; 2 College of Health and Life Sciences (CHLS), Hamad Bin Khalifa University (HBKU), Qatar Foundation (QF), Doha, Qatar; 3 Present Address: Biological Sciences, Carnegie Mellon University- Qatar, Doha, Qatar

**Keywords:** LDHC, Breast cancer, Immune checkpoint, Cytokines, Chemokines

## Abstract

**Background:**

Immunotherapy has significantly improved outcomes for cancer patients; however, its clinical benefits vary among patients and its efficacy across breast cancer subtypes remains unclear. To enhance immunotherapy efficacy, it is important to gain more insight into tumor-intrinsic immunomodulatory factors that could serve as therapeutic targets. We previously identified Lactate Dehydrogenase C (LDHC) as a promising anti-cancer target due to its role in regulating cancer cell genomic integrity. In this study, we investigated the effects of tumor LDHC expression on immune responses.

**Methods:**

TIMER AND TIDE deconvolution methods were used to investigate the relationship between tumor *LDHC* expression, immune cell infiltration and T cell dysfunction. Multiplex cytokine assays and flow cytometry were used to assess the effect of *LDHC* knockdown on the secretion of inflammatory molecules and expression of immune checkpoint molecules in breast cancer cells and cancer cell-immune cell co-cultures. T cell activity was determined by IFN-γ ELISPot assays and 7-AAD flow cytometry.

**Results:**

TIMER and TIDE analyses revealed that tumor *LDHC* expression is associated with T cell dysfunction in breast cancer and poorer post-immunotherapy survival in melanoma. Silencing *LDHC* in breast cancer cell lines (MDA-MB-468, BT-549, HCC-1954) enhanced early T cell activation and cytolytic activity. To gain a better understanding of the underlying mechanisms, comparative analysis of the effects of *LDHC* knockdown in cancer cell monocultures and co-cultures was conducted. Following *LDHC* knockdown, we observed an increase in the secretion of tumor-derived pro-inflammatory cytokines (IFN-γ, GM-CSF, MCP-1, CXCL1), a decrease in the soluble levels of tumor-derived immunosuppressive factors (IL-6, Gal-9) and reduced tumor cell surface PD-L1 expression. In direct co-cultures, *LDHC* knockdown reduced the levels of pro-tumorigenic cytokines (IL-1β, IL-4 and IL-6) and increased the secretion of the chemokine CXCL1. In addition, the number of CD8 + T cells expressing PD-1 and CTLA-4 and the cell surface expression of CTLA-4, TIGIT, TIM3, and VISTA were reduced.

**Conclusions:**

Our findings suggest that targeting LDHC could enhance anti-tumor immune responses by modulating cytokine and chemokine secretion in addition to impairing immune checkpoint signaling. Further studies are required to elucidate the molecular mechanisms by which LDHC modulates immune responses in breast cancer.

**Supplementary Information:**

The online version contains supplementary material available at 10.1186/s12964-025-02139-6.

## Background

Tumor development and progression is driven by a complex interplay of genetic, environmental and lifestyle factors that drive cellular transformation, shape the tumor microenvironment and impact the anti-tumor immune response. Under normal physiological conditions, the innate and adaptive immune responses coordinate to detect and eliminate threats, including transformed cells. However, as tumors evolve, they enter the escape phase of immunoediting, where tumor cells develop mechanisms to evade immune detection and destruction [1]. Prior to this, tumors reside in an equilibrium state, expressing various factors that create an immunosuppressive environment and promote tumor progression. This dysregulation of the anti-tumor immune response is linked to poor prognosis in many solid cancers, spurring the development of immunotherapies aimed at reprogramming and reinvigorating the host immune response to recognize and kill tumor cells [2]. In particular, immune checkpoint blockade, which targets inhibitory receptors such as CTLA-4 and PD-1/PD-L1, has shown significant clinical benefit in specific cancer types such as melanoma and non-small cell lung cancer [3, 4]. To date, eight immune checkpoint inhibitors targeting CTLA-4 (ipilimumab), PD-1 (pembrolizumab, nivolumab, cemiplimab, dostarlimab), and PD-L1 (atezolizumab, durvalumab, avelumab) have received FDA approval for the treatment of unresectable or metastatic solid tumors, including triple negative breast tumors [5, 6]. Despite their ability to induce durable responses, the majority of patients do not respond favorably to immune checkpoint blockade due to various immune evasion mechanisms [7]. This highlights the need for combinatorial strategies that can improve the efficacy of immune checkpoint inhibitors. For example, targeting tumor-intrinsic oncogenic mediators that contribute to immunosuppression could help to significantly improve the efficacy of immunotherapy.

Cancer testis antigens (CTAs) have gained attention as promising tumor antigens for therapeutic targeting. They form a class of proteins that exhibit either a testis-restricted (expressed only in testis) or testis-selective (expressed in testis and up to two other tissues) expression, and are often aberrantly expressed in cancer tissues [8, 9]. This highly-tumor restricted expression, along with their immunogenic properties and multifaceted roles in oncogenic processes, makes CTAs excellent candidates for therapeutic targeting with minimal adverse side effects. Lactate dehydrogenase C (LDHC, LDHX, CT32) is a CTA that has been shown to be aberrantly expressed in several solid cancers, including breast, lung, renal and colon cancer, and exhibits immunogenic properties [8, 10]. LDHC plays a critical role in regulating sperm energy metabolism and motility through the interconversion of pyruvate and lactate [11, 12]. In tumor cells, LDHC contributes to metabolic reprogramming by driving aerobic glycolysis and promotes tumor cell invasion, migration, and proliferation through activation of the PI3K/Akt/GSK-3B signaling pathway [13, 14]. Tumor LDHC expression is regulated by the Sp1 and CREB transcription factors, in addition to promoter CpG island hypomethylation [15]. Circulating levels of LDHC in serum and serum-derived exosomes have been shown to positively correlate with cancer diagnosis, tumor size, and recurrence in breast and hepatocellular carcinoma [16, 17]. Furthermore, *LDHC* expression in renal tumor tissue is associated with shorter survival, further supporting its role in tumor progression and poor clinical outcome [18]. Moreover, we recently demonstrated that silencing of *LDHC* in breast cancer cells leads to DNA damage accumulation and dysregulation of the cell cycle, impairing clonogenic ability [19]. Hence, LDHC can be categorized alongside PBK, SSX2 and MAGE-C2 as one of the 27 CTAs involved in regulating genomic integrity [20].

In this study, we explored the role of LDHC expression in tumor cells in the tumor immune microenvironment, particularly in the intricate dynamic interplay between tumor cells and immune cells that drive immune escape. Previously, we observed an increased expression of *LDHC* in basal-like and Her2-enriched breast tumors compared to the less aggressive luminal and normal-like subtypes [19], suggesting that LDHC may play a more prominent role in basal-like or Her2-enriched tumors, which are associated with more immunogenic tumor microenvironments [21]. To further investigate this, we examined whether LDHC expression in basal-like and Her2-enriched breast cancer cells impacts immune cell function. Utilizing co-culture models, we found that LDHC expression in breast cancer cells likely promotes an immunosuppressive tumor microenvironment, impairing immune cell function through alterations in cytokine secretion and modulation of immune checkpoint expression. In our previous work, we demonstrated that aberrant expression of PReferentially expressed Antigen in Melanoma (PRAME) in tumor cells contributes to dampening the adaptive immune response [22]. This suggests that at least two CTAs—LDHC and PRAME – play vital roles in tumor development and progression by regulating anti-tumor immune responses. Our findings indicate that targeting cancer testis antigens such as LDHC and PRAME could provide novel approaches to both disrupt pro-tumorigenic intrinsic mechanisms and overcome immune evasion. Thus, we advocate for the development of combinatorial therapies, targeting CTAs in combination with immune-based interventions, to enhance the effectiveness of current immunotherapies and improve patient outcomes.

## Methods

### Cell culture

MDA-MB-468, HCC-1954 and BT-549 cell lines were purchased from the American Tissue Culture Collection (ATCC). MDA-MB-468 cells were maintained in Dulbecco’s Modified Eagle’s Medium (Gibco-BRL) supplemented with 10% (v/v) Fetal Bovine Serum (FBS) (Hyclone US origin, GE Lifescience), 50 U/ml penicillin and 50 μg/ml streptomycin (Gibco-BRL). HCC-1954 cells were maintained in ATCC-formulated Roswell Park Memorial Institute (RPMI) 1640 medium (Gibco-BRL) supplemented with 10% (v/v) FBS, 50 U/ml penicillin and 50 μg/ml streptomycin. BT-549 cells were maintained in ATCC-formulated RPMI 1640 medium supplemented with 10% (v/v) FBS, 50 U/ml penicillin and 50 μg/ml streptomycin (Gibco-BRL), and 0.023 IU/ml insulin (Sigma-Aldrich). All cell lines were maintained in a humidified incubator at 37 °C, 5% CO2 and regular mycoplasma testing was performed using a PCR-based assay.

### *LDHC* silencing

LDHC siRNA #1–4 smartpool (siGENOME SMARTpool) and control siRNA #1–4 smartpool (siGENOME non-targeting siRNA Pool#1, D-001206–13-20) were purchased from Dharmacon. Adherent cancer cells were transfected at 60–70% confluency with 20 nM siRNA pools and Lipofectamine RNAiMAX (Thermo Fisher Scientific) following the manufacturer’s instructions. Knockdown of *LDHC* expression was assessed by real time qRT-PCR and western blotting.

### RNA extraction and quality assessment

Total RNA was isolated using the PureLink RNA Mini kit (Ambion) following the manufacturer’s protocol. The RNA quantity and purity were assessed by Nanodrop measurement. Reverse transcription of 1 µg RNA was performed using MMLV-Superscript reverse transcriptase (Thermo Fisher Scientific) and random hexamers (Applied Biosystems) resulting in a final concentration of 50 ng/µl cDNA.

### Quantitative real-time reverse transcription polymerase chain reaction

*LDHC* expression was quantified using specific 5′FAM-3′MGB Taqman gene expression primer/probe sets (Hs00255650_m1, Applied Biosystems). *PD-L1*, *CD80*, *GAL-9* and *PVR* expression was quantified by SYBR Green-based qPCR using PowerUp SYBR Green mastermix (Applied Biosystems) and primers designed through PrimerBLAST (NCBI). qRT-PCR was performed on the QuantStudio 7 system (Applied Biosystems). Relative expression levels were normalized to the housekeeping gene RPLPO using a Taqman gene expression primer/probe set (4333761F, Applied Biosystems) or primers designed for SYBR-Green based PCR (Forward primer sequence 5’-TCCTCGTGGAAGTGACATCG-3’, Reverse primer sequence 5-TGGATGATCTTAAGGAAGTAGTTGG-3’).

### Western blotting

Cell protein lysate was isolated using RIPA buffer (Pierce) supplemented with HALT protease and phosphatase inhibitor cocktail (Thermo Fisher Scientific). Western blotting was performed using a standard protocol as previously described [23]. Primary antibodies utilized include antibodies against LDHC (ab52747, Abcam, 1:500), PD-L1 (#13684, Cell Signaling Technologies, 1:1000) and β-actin (#4970, Cell Signaling Technologies, 1:1000). Horseradish Peroxidase (HRP)-linked anti-rabbit/mouse secondary antibody incubation followed by enhanced chemiluminescent substrate (ECL) Supersignal West Femto (Pierce) incubation was used to visualize the protein bands of interest on the ChemiDoc XRS + Imaging system (Biorad).

### Peripheral blood lymphocyte (PBL) isolation

Buffy coat samples, collected from healthy donors at the Hamad Medical Corporation Blood Donation Center, were diluted with Dulbecco's Phosphate-Buffered Saline (DPBS, Gibco-BRL), layered on Lymphoprep™ (Stem Cell Technologies) and subjected to density gradient centrifugation to isolate the peripheral blood mononuclear cells (PBMCs). The PBMCs were washed and frozen in liquid nitrogen in freezing media (50% FBS, 40% serum-free Roswell Park Memorial Institute 1640 medium (RPMI), 10% Dimethyl sulfoxide) until further use. Prior to assays, PBMCs were defrosted in RPMI medium supplemented with 10% FBS and incubated overnight at 37 °C and 5% CO2. To isolate the non-adherent peripheral blood lymphocyte population, the cells were plated in a flat-bottom multi-well plate (Thermo Fisher Scientific, Nunclon Δ Surface) and incubated for 2 h at 37 °C and 5% CO2. Next, the non-adherent PBLs were activated overnight using 2 μg/ml of plate-bound anti-human CD3 and CD28 antibodies (eBioscience) at 37 °C and 5% CO2. HLA typing of PBMCs was obtained for nine different HLA loci (A, B, C, DRB1, DRB3/4/5, DQA1, DQB1, DPA1, and DPB1) with one or two field resolution and donors with matched loci to MDA-MB-468, BT-549 and HCC-1954 cancer cells were selected for further analysis.

### Indirect co-culture

A total of 5 × 10^4^ cancer cells were seeded per well in a 24-well plate. Next, activated PBLs were placed on top using transwell inserts with 0.4 µm pore size (Corning) and a Target:Effector (T:E) ratio of 1:20 to enable exchange of soluble factors between cancer cells and PBLs without direct cell–cell contact. Wells with PBLs alone were used as a negative control. The cells were co-cultured for 72 h at 37 °C and 5% CO2, after which the PBLs, cancer cells and conditioned media were collected for flow cytometry and cytokine multiplex analysis.

### Direct co-culture

Activated PBLs were seeded at 1 × 10^6^ cells per well in a U-bottom 96-well plate. Cancer cells were labelled with 10 nM Qtracker™ − 655 (Thermo Fisher Scientific) according to manufacturer's instructions. A total of 2 × 10^4^ cancer cells were added to the wells with activated PBLs (T:E ratio 1:50) and co-cultured at 37 °C, 5% CO2. After 4 h of direct co-culture, the cells were harvested for interferon (IFN)-γ ELISpot assay and cytotoxicity analysis by flow cytometry. In addition, 72 h direct co-cultures of cancer cells and PBLs at T:E ratio of 1:20 were performed, and cells and conditioned media were collected for immune checkpoint flow cytometry and cytokine multiplex analysis.

### IFN-γ ELISpot

The Human IFN-γ ELISpot PLUS kit (HRP, Mabtech) was utilized according to manufacturer's instructions. A total of 5 × 10^4^ PBLs from short-term direct (4 h) co-culture experiments were seeded per well. PBLs activated with 2 μg/ml of plate-bound anti-human CD3 and CD28 antibodies (eBioscience) or PBS served as positive and negative controls respectively. The wells were incubated at 37 °C, 5% CO2 for 24 h and the number of IFN-γ spot forming units were quantified using an ELISpot reader (Autoimmun Diagnostika GmbH).

### Cytotoxicity analysis

After 4 h direct co-culture, cells were washed and resuspended in PBS, followed by staining with 7-Aminoactinomycin D (7-AAD) (eBiocience) at room temperature for 5 min. Flow cytometry was performed by recording 50,000 events/sample using the LSRFortessa X-20 instrument and FlowJo V10.10.0 software (BD Biosciences). Non-viable cancer cells were gated as positive for Qtracker and 7-AAD staining.

### Cytokine multiplex analysis

Cell culture supernatants from 72 h direct co-cultures were diluted 1:2 and used to determine the expression of 23 soluble proteins with the Bio-Plex Pro Human Cytokine 17-plex Assay (Biorad #M5000031YV) and a custom 6-plex Luminex array (R&D Systems, Table S1) according to manufacturer’s instructions using the Bioplex-200 system (Biorad). A 13-point standard curve was generated to extrapolate the soluble protein levels and the data was analyzed using the Bioplex Manager Software (Biorad).

### Flow cytometric analysis of immune checkpoint expression

PBLs and cancer cells from direct and indirect co-cultures were used for multi-marker flow cytometry to detect the expression of immune checkpoint markers. Cells were washed and resuspended in 100 μl of staining buffer containing Human Fc Block™ (564,219, BD Biosciences). The expression of immune checkpoint receptors was determined using the following antibodies: PD-1 PE-Dazzle 594 (329,940, Biolegend), VISTA BV421 (566,750, BD Biosciences), CTLA-4 BV786 (563,931, BD Biosciences), TIM3 BV650 (565,565, BD Biosciences), LAG-3 PE (565,617, BD Biosciences), TIGIT BUV395 (747,845, BD Biosciences) and CD8 BV510 (563,919, BD Biosciences). In parallel, we determined the expression of respective ligands: CD80 BV510 (740,150, BD Biosciences), CD86 Alexa700 (564,544, BD Biosciences), PD-L1 PE-Cy7 (558,017, BD Biosciences), PD-L2 BV786 (563,843, BD Biosciences), VISTA BV421 (566,750, BD Biosciences), HLA-DR BV650 (564,231, BD Biosciences), GAL-9 PE (565,890, BD Biosciences) and PVR BUV395 (748,272, BD Biosciences). Flow cytometry was performed by recording 50,000 events/sample using the LSRFortessa X-20 instrument and FlowJo V10.10.0 software (BD Biosciences). Gating strategies are depicted in Fig S1 and Fig S2.

### Tumor immune infiltration, dysfunction and exclusion analyses

The Tumor IMmune Estimation Resource (TIMER) algorithm [24] was applied to investigate the relationship between tumor *LDHC* expression and immune cell infiltration (http://timer.cistrome.org/). In addition, we used the TIMER 2.0 platform to apply the Estimating the Proportions of Immune and Cancer cells (EPIC) algorithm which generates direct estimates of cell proportions by fitting single-cell RNA expression data to bulk RNAseq data. Finally, we utilized the Tumor Immune Dysfunction and Exclusion (TIDE) algorithm [25, 26] to predict the effect of tumor *LDHC* expression on T cell dysfunction and immunotherapy response (http://tide.dfci.harvard.edu).

### Protein–protein interaction network and gene ontology analysis

Protein–protein interaction network analysis was performed using STRING online tool (http://www.string-db.org), and visualization was achieved using full STRING network with high confidence network edges. Gene ontology (GO) analysis was conducted using the Enrichr tool [27–29] and GO terms, involving multiple genes, were visualized using SRplot [30].

### Statistical analyses

Statistical analyses were performed using the Student’s t-test with p values ≤ 0.05 deemed being statistically significant. Data of at least three independent biological replicates are represented as mean ± standard error of mean (SEM), unless stated otherwise. Statistical analyses and data visualization were performed using GraphPad Prism v10.0.0.

## Results

### Aberrant tumor *LDHC* expression is associated with T cell dysfunction.

**Fig. 1:**
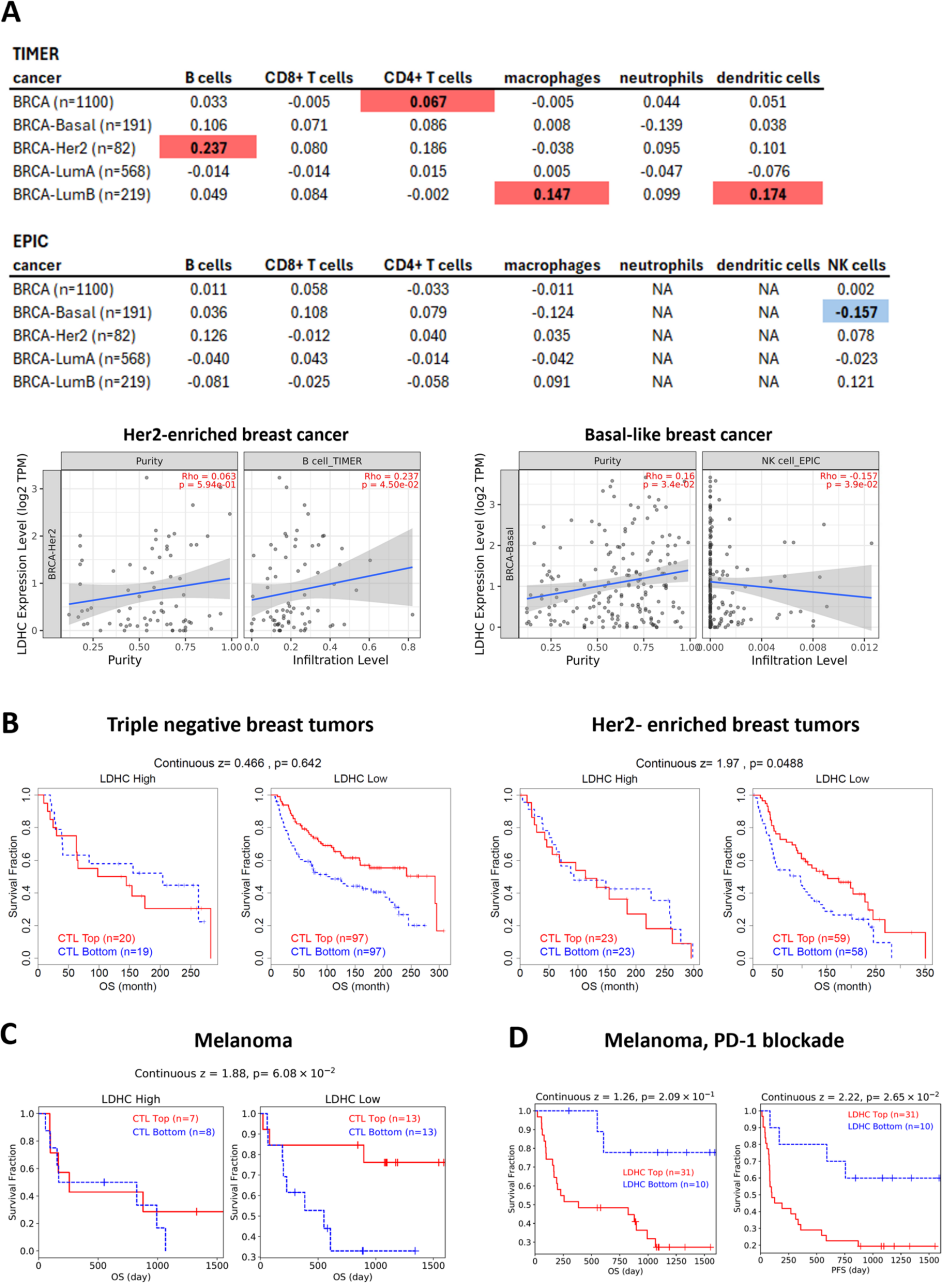
Association of tumor *LDHC* expression with immune cell infiltration and T cell dysfunction. **A** Correlation of tumor *LDHC* expression and infiltration of immune cells subsets according to TIMER and EPIC deconvolution methods. Significant positive and negative spearman's rho values are highlighted in red and blue respectively. Significant correlations in Her2-enriched and basal-like breast cancer are depicted in scatter plots. **B** Kaplan Meier plots of triple negative and Her2-enriched breast cancer patients classified as having *LDHC* high or low expressing tumors and dichotomized by cytotoxic T lymphocyte (CTL) infiltration level. **C** Survival analysis of melanoma patients with *LDHC* high or low expressing tumors by CTL level. **D** Kaplan Meier survival curves of melanoma patients receiving PD-1 blockade, illustrating overall and progression-free survival in relation to tumor *LDHC* expression

We investigated the relationship between aberrant *LDHC* tumor expression, immune cell infiltration, and T cell function in breast cancer using various deconvolution methods. To gain more insight into potential associations between tumor *LDHC* expression and immune cell infiltration, we applied the Tumor IMmune Estimation Resource (TIMER) algorithm to estimate the relative abundance of six major immune cell types (B cells, CD8 + T cells, CD4 + T cells, macrophages, neutrophils and dendritic cells) in the TCGA breast cancer dataset (Fig. 1A). We observed a positive correlation between *LDHC* expression and B cell infiltration in Her2-enriched tumors, macrophages and dendritic cells in luminal B tumors, and CD4 + T cells across all breast cancer samples. Next, we used the Estimating the Proportions of Immune and Cancer cells (EPIC) algorithm to account for tumor purity variability across samples, providing a more accurate direct estimate of cell proportions within tumors. Using EPIC, we did not find significant associations between *LDHC* expression and the infiltration of the six major immune cell subtypes previously analyzed with TIMER. However, we did find a negative correlation between *LDHC* expression and NK cell infiltration in basal-like breast tumors, a cell type which is not available for TIMER analysis. Considering both methods, we found that *LDHC* expression was negatively associated with NK cell infiltration in basal-like breast tumors and positively correlated with B cell infiltration in Her2-enriched breast tumors. Both methods also indicated that *LDHC* expression was positively correlated with tumor purity, suggesting that tumor cells are likely the main source of LDHC in the tumor microenvironment (Fig. 1A—bottom). To investigate whether LDHC expression affects immune function and impacts clinical outcomes or responses to immunotherapy, we used the Tumor Immune Dysfunction and Exclusion (TIDE) algorithm. TIDE classifies tumors with high or low *LDHC* expression into two groups based on cytotoxic T lymphocyte (CTL) abundance. While high CTL infiltration is commonly associated with good prognosis in cancer, we observed that high expression of *LDHC* in Her2-enriched and triple negative breast tumors reduced or negated the favorable association between CTL infiltration and overall survival, suggestive of T cell dysfunction that could negatively impact immunotherapy responses (Fig. 1B). Given the lack of immunotherapy response data for breast cancer in the TIDE database, we explored the effect of *LDHC* expression on treatment response in melanoma patients receiving PD-1 immune checkpoint blockade thereapy. Similar to our observations in Her2-enriched and triple negative breast cancer, high *LDHC* expression in melanoma significantly diminished the favorable association between CTL infiltration and overall survival (Fig. 1C). Moreover, melanoma patients with high *LDHC* tumor expression exhibited shorter overall and relapse-free survival in response to PD-1 blockade (Fig. 1D). These findings suggest that while aberrant tumor expression of *LDHC* may not strongly affect immune cell infiltration, it is associated with T cell dysfunction.

### Silencing of *LDHC* enhances T cell activation and cytolytic function

**Fig. 2:**
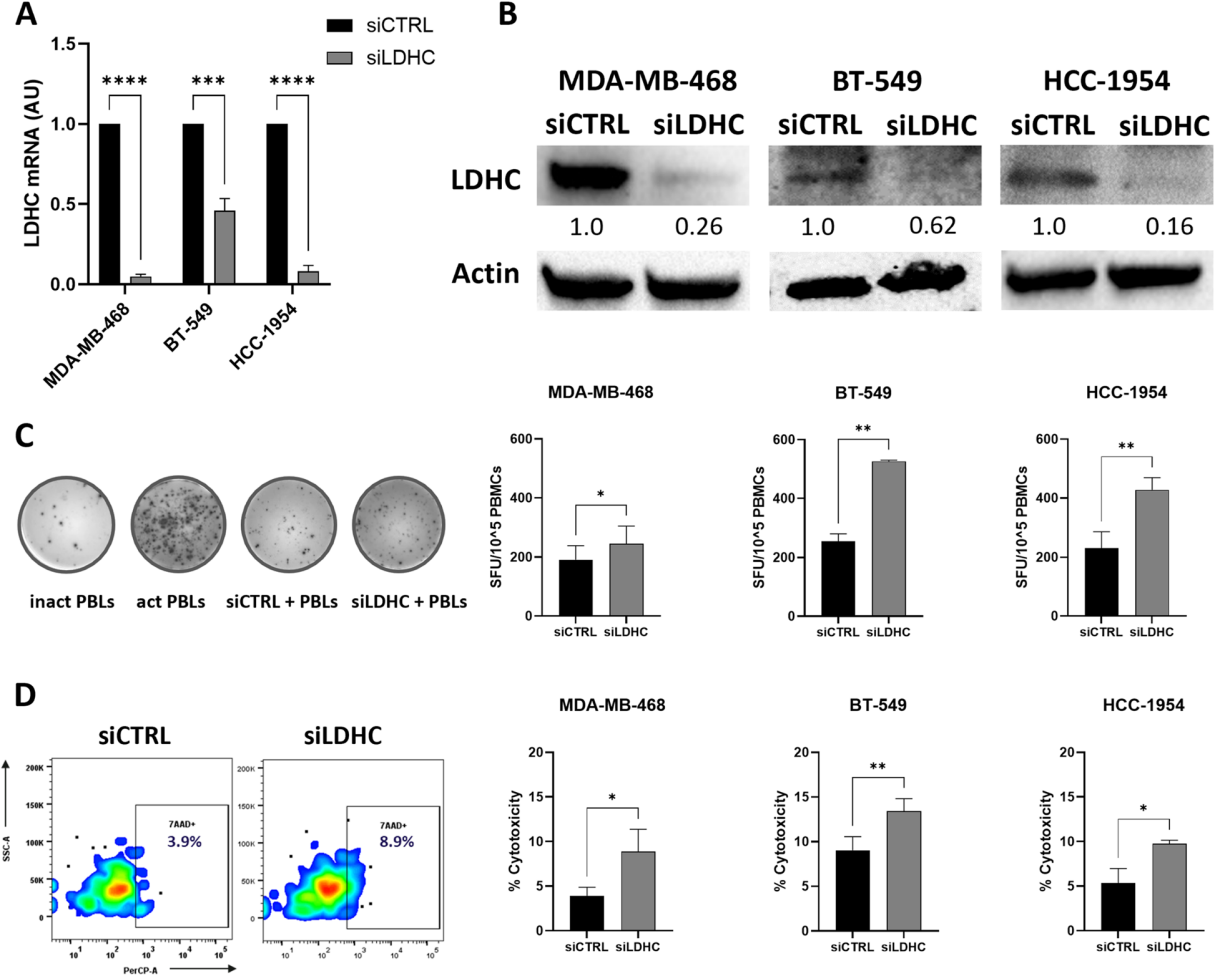
*LDHC* knockdown enhances T cell function and cytolytic activity. **A** Validation of *LDHC* silencing efficiency in three breast cancer cell lines using real-time qRT-PCR (normalized to expression of housekeeping gene *RPLPO*). Bar charts are representative of combined data from 3 independent experiments. Statistical analysis comparing siCTRL vs siLDHC performed using unpaired Student's t-test. **B** Confirmation of *LDHC* knockdown by western blotting. Representative image from three independent experiments. **C** Analysis of T cell activity as measured by IFN-γ ELISpot after short-term direct co-culture of cancer cells with HLA-matched peripheral blood lymphocytes. Combined data from 3 independent experiments, each performed with one donor (biological replicate), and one representative image (MDA-MB-468 cells) are depicted. **D** Cell viability flow cytometry analysis after short-term direct co-culture. Non-viable cancer cells were gated as positive for Qtracker and 7-AAD staining. Combined data from 3 independent experiments, each performed with one donor (biological replicate), and one representative flow cytometry plot (MDA-MB-468 cells) are shown. Bar charts represent mean with standard error of mean (± SEM) from three independent replicates. *p ≤ 0.05, **p ≤ 0.01, ***p ≤ 0.001, **** *p* ≤ 0.0001

To investigate the role of LDHC tumor expression in regulating T cell function, we generated breast cancer cell models with varying levels of LDHC expression by knocking down *LDHC* in two basal-like cell lines (MDA-MB-468, BT-549) and one Her2-enriched cell line (HCC-1954). Downregulation of LDHC expression was confirmed both at the RNA and protein level in all three cell lines (Fig. 2A-B). Next, we co-cultured *LDHC*-silenced tumor cells and their control counterparts with peripheral blood lymphocytes and assessed IFN-γ secretion using the ELISpot assay. Silencing of *LDHC* significantly increased IFN-γ secretion, a marker of T cell activation, in all three cell lines (Fig. 2C). In addition, we observed a significant increase in immune cell-mediated cancer cell killing following *LDHC* silencing in all cell lines (Fig. 2D).

### Tumor *LDHC* expression alters the production of tumor-derived inflammatory mediators and expression of immune checkpoint ligands

**Fig. 3:**
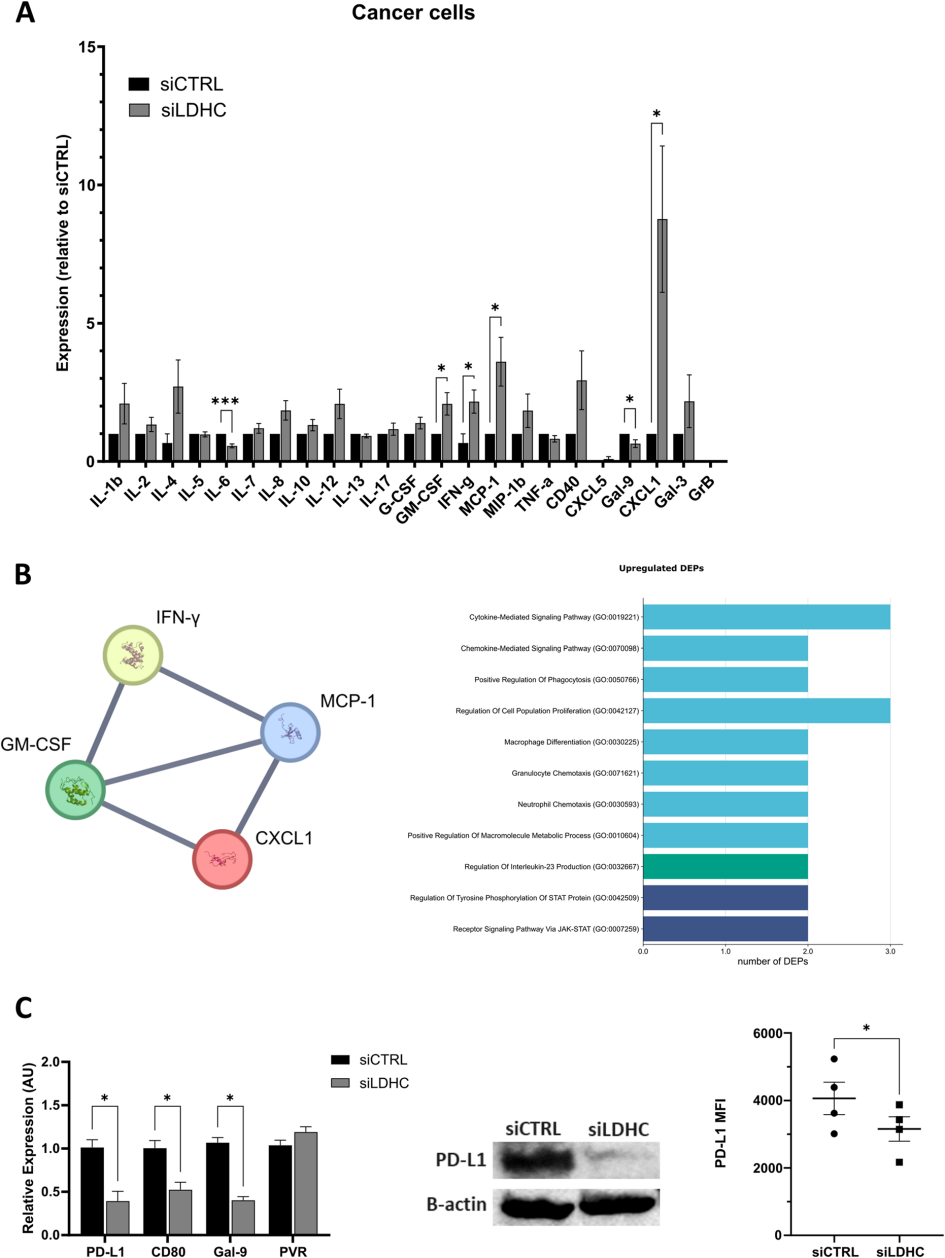
*LDHC* knockdown alters tumor-derived cytokine and chemokine secretion and immune checkpoint expression in MDA-MB-468 breast cancer cells. **A** Cytokine and chemokine levels measured by 23-plex using 72-h conditioned media from cancer cells following *LDHC* knockdown. Protein levels are expressed as mean fold-change relative to siCTRL. **B** Protein–protein interaction network and gene ontology analysis of proteins with increased expression in *LDHC*-silenced cells. **C** Analysis of immune checkpoint expressions using real time qRT-PCR, western blotting and flow cytometry. RNA expression data was normalized to *RPLPO* expression and plotted as mean fold-change relative to siCTRL. For western blotting, β-actin protein expression was used as a loading control. Data is representative of a minimum of three independent experiments. Bar charts represent mean with standard error of mean (± SEM). Statistical analysis performed using Student's t-test. * *p* ≤ 0.05, *** *p* ≤ 0.001. MFI, mean fluorescence intensity; DEP, differentially expressed protein

Following our observation that decreased LDHC expression in breast cancer cells was associated with enhanced T cell functionality, we performed an in-depth analysis of LDHC-associated changes in immune-related molecules. For these analyses, we utilized the basal-like MDA-MB-468 breast cancer cell line, which demonstrated robust *LDHC* knockdown efficiency and the highest increase in T cell-mediated cancer cell cytotoxicity following *LDHC* silencing. Using a 23-plex bead-based immunoassay, we found that knockdown of *LDHC* significantly increased the levels of cancer cell-derived GM-CSF, IFN-γ, MCP-1, and CXCL1, while reducing IL-6 and Gal-9 levels (Fig. 3A). Protein–protein interaction network analysis (Fig. 3B) revealed that the upregulated proteins form a high-confidence network (PPI enrichment p value = 2.5e-8). Gene ontology analysis indicated that these upregulated molecules are mainly involved in processes supporting an immune favorable environment, including cytokine and chemokine-mediated signaling, phagocytosis, cell proliferation, macrophage differentiation, chemotaxis and STAT signaling (Fig. 3B). Furthermore, the increase in IFN- γ levels is in accordance with the enhanced T cell activation and cytotoxic activity we observed in direct co-cultures (Fig. 2C-D). In turn, the reduction in soluble IL-6 and Gal-9 levels likely contributes to improved anti-tumor immunity by alleviating the pro-tumorigenic and immunosuppressive effects of IL-6 and by disrupting the interaction between Gal-9 and the immune checkpoint receptor TIM3 on T cells.

In addition to soluble inflammatory molecules, stimulatory and inhibitory immune checkpoints play a critical role in regulating anti-tumor immunity. Hence, we assessed the tumor cell expression of four distinct immune checkpoint ligands; *PD-L1*, *CD80*, *GAL-9,* and *PVR*, which interact with the clinically relevant receptors *PD-1*, *CTLA-4, TIM3*, and *TIGIT* on T cells. Silencing *LDHC* significantly downregulated the mRNA expression of *PD-L1*, *CD80* and *GAL-9* in MDA-MB-468 breast cancer cells (Fig. 3C). Further analysis of BT-549 and HCC-1954 demonstrated a significant downregulation of *PD-L1* expression in both cell lines, with a trend towards reduced *GAL-9* expression in HCC-1954 cells (Fig S3). Given the clinical significance of PD-1/PD-L1 blockade in multiple cancers, we further validated the downregulation of PD-L1 in *LDHC*-silenced MDA-MB-468 cells using western blotting and flow cytometry (Fig. 3C).

### Immunomodulatory effects of aberrant *LDHC* tumor expression require direct cell–cell contact of cancer cells with immune cells

**Fig. 4:**
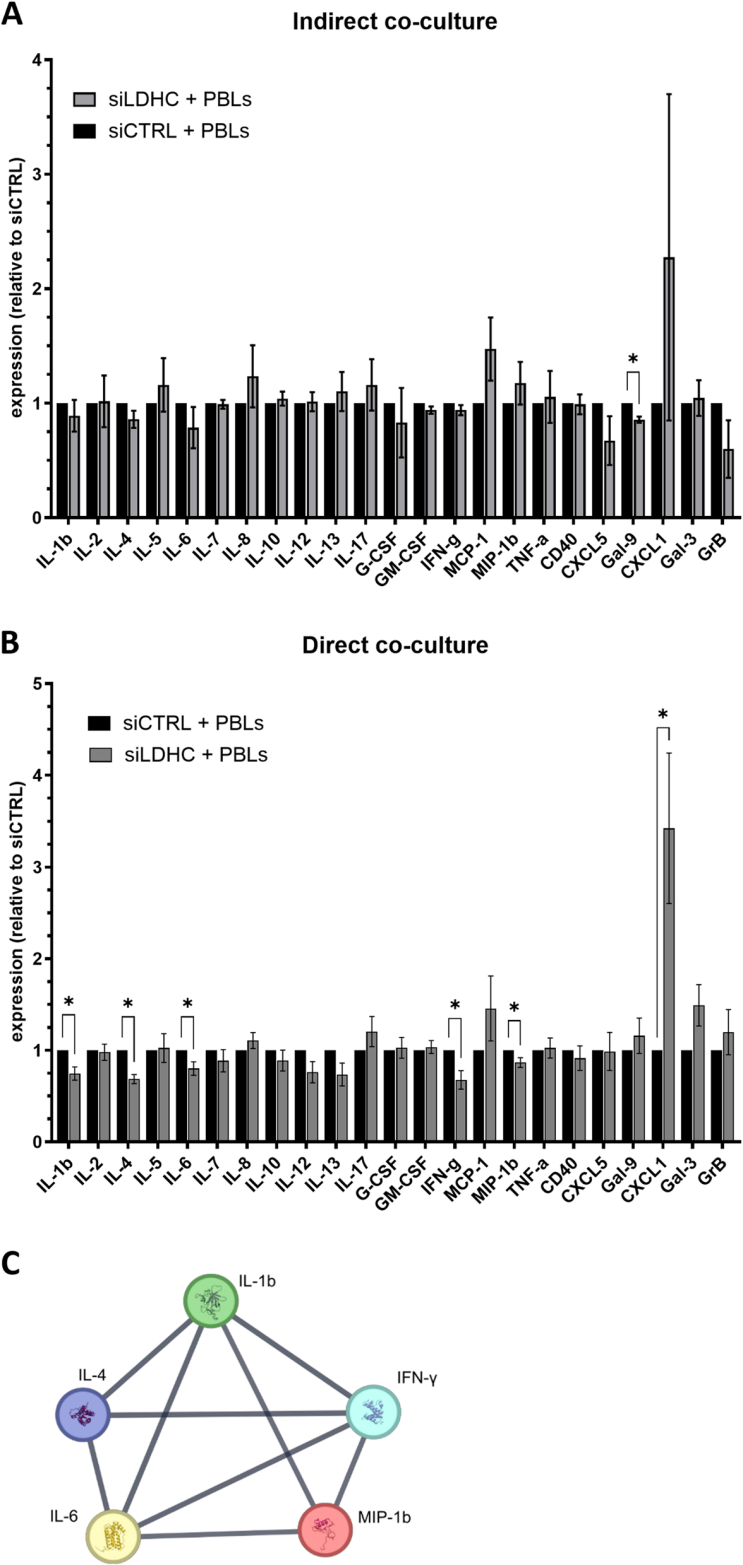
*LDHC* knockdown in MDA-MB-468 breast cancer cells alters soluble cytokine and chemokine levels in indirect and direct co-cultures. **A** Cytokine and chemokine levels measured by 23-plex using 72 h conditioned media from indirect co-cultures. **B** Cytokine and chemokine analysis of 72-h conditioned media from direct cocultures. **C** Protein–protein interaction network of downregulated proteins in *LDHC*-silenced cells. Protein levels are expressed as mean fold-change relative to siCTRL. Combined data from a minimum of three independent experiments, each performed with one donor (biological replicate), are shown. Bars indicate mean with standard error of mean (± SEM). Statistical analysis performed using unpaired Student's t-test. * *p* ≤ 0.05

Since we found that aberrant expression of LDHC in tumor cells impacts T cell activity (Fig. 2C-D) and dysregulates the secretion of tumor-derived pro-inflammatory molecules (Fig. 3A-B), we sought to assess the effects of *LDHC* silencing on cytokine levels in cancer cell-PBL co-culture models. Using an indirect co-culture model, we found a mere decrease in Gal-9 levels (*p* < 0.01), similar to what we observed in cancer cell monocultures (Fig. 4A). Using direct co-culture of *LDHC*-silenced cancer cells with PBLs, we observed multiple changes including a significant decrease in the pro-tumorigenic cytokines IL-1β (*p* < 0.01), IL-4 (*p* < 0.01), and IL-6 (*p* = 0.03), alongside IFN-γ (*p* = 0.01) and MIP-1b (*p* = 0.04) and an increase in CXCL1 (*p* = 0.04) (Fig. 4B). Network analysis revealed high-confidence interconnections (PPI enrichment p value = 1.21e-12) between each of the five downregulated molecules (Fig. 4C).

**Fig. 5:**
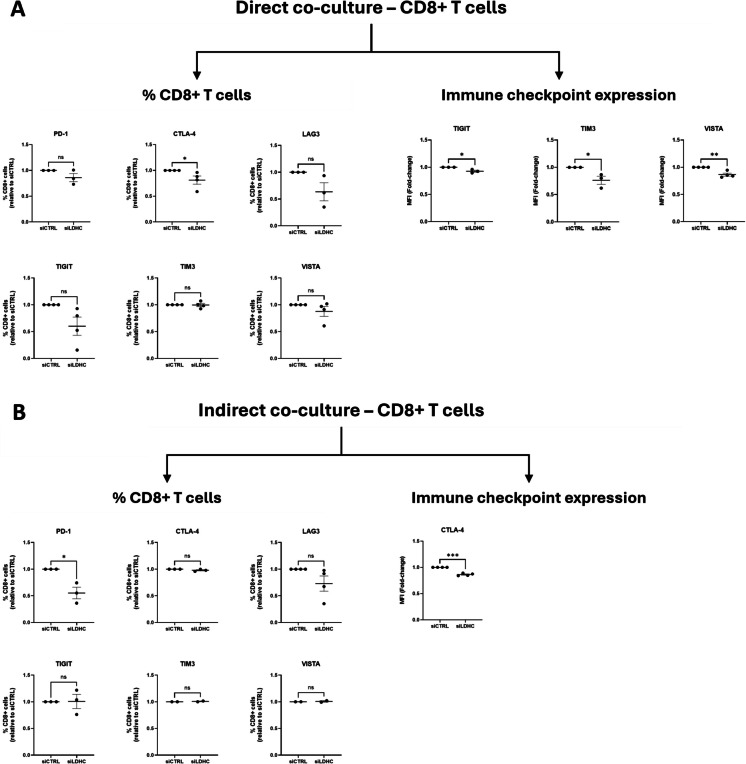
*LDHC* knockdown reduces expression of immune checkpoint receptors on CD8 + T cells following direct and indirect coculture with MDA-MB-468 breast cancer cells. **A** Flow cytometry analysis to assess the frequency of CD8 + T cells expressing PD-1, CTLA-4, LAG-3, TIGIT, TIM3 and VISTA after 72 h of direct co-culture *(left)*, and their expression of TIGIT, TIM3 and VISTA *(right)*. **B** Flow cytometry analysis of the number of CD8 + T cells expressing immune checkpoint receptors after 72 h of indirect co-culture *(left)* and of CTLA-4 expression *(right)*. Dot plots represent mean fold-change relative to siCTRL with standard error of mean (± SEM). Combined data from multiple independent experiments, each performed with one PBL donor (biological replicate), are shown. Statistical analysis performed using paired Student's t-test. * *p* ≤ 0.05, ** *p* ≤ 0.01, *** *p* ≤ 0.001

**Fig. 6:**
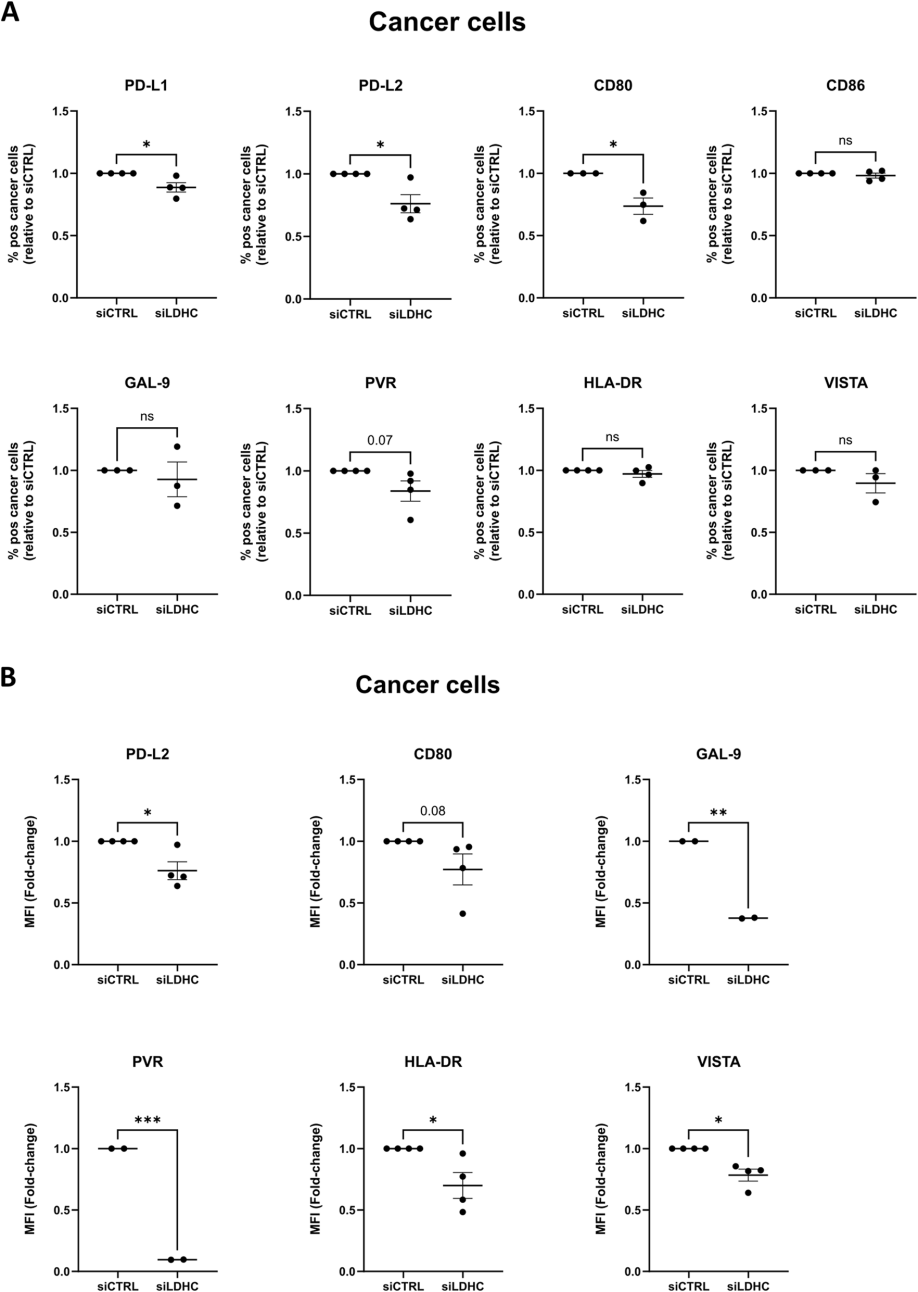
*LDHC* knockdown reduces expression of immune checkpoint ligands on MDA-MB-468 cancer cells following indirect co-culture. **A** Flow cytometry analysis of the number of cancer cells expressing PD-L1, PD-L2, CD80, CD86, GAL-9, PVR, HLA-DR and VISTA after 72 h of indirect co-culture. **B** Flow cytometry analysis of the expression of immune checkpoint ligands on cancer cells. Dot plots represent mean fold-change relative to siCTRL with standard error of mean (± SEM). Combined data from multiple independent experiments, each performed with one PBL donor (biological replicate), are shown. Statistical analysis performed using paired Student's t-test. * *p* ≤ 0.05, ** *p* ≤ 0.01, *** *p* ≤ 0.001

Furthermore, we assessed the expression of immune checkpoint ligands and receptors. Analysis of CD8 + T cell surface expression revealed a significant reduction in the number of cells expressing CTLA-4 in direct co-cultures and PD-1 in indirect co-cultures (Fig. 5A-B). Additionally, the expression of immune checkpoint molecules on the T cells was altered upon co-culture. For instance, TIGIT, TIM3 and VISTA expression were downregulated in direct co-cultures with *LDHC-*silenced cancer cells by 72 h, while CTLA-4 expression was reduced in indirect co-cultures (Fig. 5A-B, Fig S4A-B). Looking at the cancer cells, we observed a reduction in the number of *LDHC*-silenced cancer cells expressing PD-L1, PD-L2 and CD80, and decrease in the cell surface expression of PD-L2, Gal-9, PVR, HLA-DR and VISTA (Fig. 6A-B, Fig S4C). Together, these findings suggest that silencing of *LDHC* in cancer cells disrupts multiple immune checkpoint signaling axes, resulting in enhanced T cell activity.

## Discussion

Traditionally, cancer treatment has focused on reducing or eliminating tumor burden through surgery, radiation therapy and chemotherapy. However, advances in cancer biology and immunology have revolutionized cancer treatment, facilitating the integration of targeted therapies and immunotherapy alongside traditional treatment approaches. In particular, immune checkpoint inhibitors targeting PD-1/PD-L1 and CTLA-4 have significantly improved clinical outcomes in patients with melanoma, non-small cell lung cancer, and renal cell carcinoma [31–33]. Despite these successes, the benefit of immunotherapy remains limited to a subset of patients, with many either failing to respond or developing severe immune-related adverse events [34]. To enhance immunotherapy efficacy, a deeper understanding of the complex interactions between tumor cells and their microenvironment—including stromal cells and immune cells – is essential.

In this study, we explored the potential of targeting LDHC in cancer cells to impact anti-tumor immunity, proposing a novel dual approach to both target tumor cells and enhance the immune response. We specifically focused on basal-like and Her2-enriched breast tumors as we have previously identified a higher *LDHC* expression in these tumors compared to luminal breast tumors [19]. Using TIDE analysis, we demonstrated that increased *LDHC* expression in these tumors correlates with T cell dysfunction. In accordance, silencing of *LDHC* in three distinct breast cancer cell lines enhanced T cell activation and cytolytic activity. A comprehensive analysis of 23 cytokines and chemokines revealed elevated levels of tumor-derived GM-CSF, IFN-γ, MCP-1 and CXCL1, alongside a decrease in IL-6 and Gal-9, further supporting the enhanced T cell activity observed following *LDHC* knockdown. Furthermore, we found a reduction in PD-L1 expression on the cell surface of *LDHC*-silenced tumor cells, suggesting that impairment of PD-L1/PD-1 signaling may contribute to improved T cell activation.

Analysis of cancer cell-immune cell co-cultures revealed that tumor LDHC expression differentially regulates the expression of soluble inflammatory mediators through direct cell–cell contact and indirect interactions. In direct co-cultures, *LDHC* knockdown resulted in multiple changes in cytokine levels, whereas in indirect co-cultures, only Gal-9 levels were affected. This discrepancy suggests that the immunomodulatory effects of tumor cell LDHC expression are largely mediated through direct cell–cell interactions rather than soluble factor signaling. We hypothesize that direct co-cultures may induce a broader range of cytokine changes via contact-dependent or juxtacrine signaling, such as polarized cytokine secretion at the immunological synapse, membrane-bound cytokine signaling, cytokine receptor internalization, and ligand-receptor interactions by adhesion and immune checkpoint molecules [35–37]. In our direct co-cultures, we observed an early increase in IFN-γ secretion and enhanced tumor cell killing activity, followed by a decrease in IL-1β, IL-4 and IL-6 levels alongside an increase in soluble CXCL1. To understand how these cytokine changes correlate with enhanced T cell activation and cytolytic activity, it is crucial to consider the context-dependent, pleiotropic functions of cytokines. In cancer, IL-1β, IL-4 and IL-6 are well-known for their tumor promoting effects, including enhancing tumor cell proliferation and survival, supporting cancer cell stemness, and facilitating invasion and angiogenesis through autocrine signaling or crosstalk with tumor infiltrating immune cells [38–40]. In contrast, CXCL1 has diverse roles in cancer, promoting tumor growth and metastasis while enhancing the anti-tumor immune response. Tumor CXCL1 expression has been shown to drive cancer cell proliferation, migration, invasion and angiogenesis in a CXCR2-dependent manner [41]. While CXCL1 is highly expressed in several triple negative breast cancer cell lines, CXCR2 expression is reported to be reduced compared to other breast cancer subtypes [42, 43]. Elevated CXCL1 expression has also been linked to improved tumor control through the recruitment and activation of neutrophils [44]. Therefore, further studies are needed to determine whether the increased CXCL1 levels following *LDHC* knockdown in triple negative breast cancer cells primarily impact tumor growth or immune cell infiltration and activation. We propose that in vivo studies using xenograft and syngeneic mouse models combined with CXCL1 neutralizing antibodies could provide valuable insights into the distinct roles of CXCL1 in *LDHC*-silenced triple negative breast cancer cells. Furthermore, *LDHC* knockdown resulted in a decrease in the expression of multiple immune checkpoint ligands and receptors, as well as a reduction in the number of PD-1 and CTLA-4 positive CD8 + T cells following co-culture. Notably, *LDHC* knockdown also resulted in lower levels of soluble Gal-9 in cancer cell monocultures and indirect co-cultures, which likely reduces its binding to the inhibitory immune checkpoint receptor TIM3, thereby enhancing T cell activation [45]. The precise mechanisms by which LDHC modulates immune checkpoint expression remain to be elucidated. As a key regulator of aerobic glycolysis, aberrant LDHC expression in tumors is presumed to increase the lactate levels in the tumor microenvironment. High lactate levels are well known to contribute to immunosuppression by directly inhibiting CD8 + T cell function and promoting the recruitment of immunosuppressive cells such as T regulatory cells (Tregs) and myeloid-derived suppressor cells (MDSCs) [46–48]. Furthermore, lactate-mediated activation of the GPR81 receptor has been shown to upregulate PD-L1 expression in lung cancer [49]. Conversely, Tregs in highly glycolytic tumors display increased PD-1 expression due to MCT1-mediated lactate uptake and subsequent nuclear translation of NFAT1 [50]. More recently, lactylation of histone and non-histone proteins has emerged as a key transcriptional regulatory mechanism, with evidence demonstrating increased lactylation at the PD-L1 promoter region, leading to enhanced PD-L1 expression in acute myeloid leukemia [51]. This suggests that LDHC may regulate immune checkpoint expression through similar lactate-driven mechanisms.

Together, these findings indicate that aberrant LDHC expression in tumors is associated with immune dysfunction through impaired T cell activation and modulation of immune checkpoint signaling. Although the cellular origin of the altered cytokine profiles in our co-cultures remains unclear, we speculate that the observed increase in CXCL1 and decrease in IL-6 levels are at least partially attributable to tumor-derived cytokine secretion, as indicated by our findings in both cancer monocultures and co-cultures. Future studies using stable isotope labeling by amino acids in cell culture (SILAC) could provide direct experimental evidence for their cellular origin. Additionally, co-cultures with different immune cell subpopulations could offer further insight into the interactions between LDHC-expressing tumor cells and distinct components of the immune response. Finally, in vivo xenograft models will be crucial to validate the immunomodulatory role of LDHC within a functional tumor immune microenvironment.

## Conclusions

Immunotherapy has dramatically changed cancer care; however, its effectiveness remains limited by a range of tumor-intrinsic and microenvironmental factors. Cancer cells secrete various pro-tumorigenic and immunoregulatory molecules that promote tumor progression and impede immune-mediated tumor elimination. Therefore, to improve therapeutic outcomes, a deeper understanding of the dynamic interactions between cancer cells and immune cells is essential to identify tumor cell surface markers and tumor-derived soluble factors that modulate anti-tumor immunity. Our findings suggest that targeting tumor LDHC expression could help establish a more favorable immune microenvironment, potentially enhancing responses to immunotherapy. Further research is needed to elucidate the molecular mechanisms by which LDHC regulates immune responses, including its metabolic role in promoting immunosuppression.

## Supplementary Information

Supplementary Material 1: Table S1. Composition of multiplex cytokine assays.

Supplementary Material 2: Figure S1. Expression analysis of immune checkpoint receptors on CD8 + T cells. Flow cytometry plots depicting CD8 + T cell and immune checkpoint receptor gating strategies. Representative plots of one donor

Supplementary Material 3: Figure S2. Expression analysis of immune checkpoint ligands on tumor cells. Flow cytometry plots depicting immune checkpoint ligand gating strategies. Representative plots of indirect co-culture of cancer cells with peripheral blood lymphocytes from one donor

Supplementary Material 4: Figure S3. *LDHC* knockdown reduces expression of immune checkpoint ligands on BT-549 and HCC-1954 breast cancer cells. A) *PD-L1* and B) *Gal-9* expression in BT-549 and HCC-1954 cells as measured by real time qRT-PCR. RNA expression data was normalized to *RPLPO* expression and plotted as mean fold-change relative to siCTRL. Combined data from a minimum of 3 independent experiments. Bar charts represent mean fold-change relative to siCTRL with standard error of mean (± SEM). Statistical analysis performed using paired Student's t-test. * *p* ≤ 0.05, ** *p* ≤ 0.01

Supplementary Material 5: Figure S4. *LDHC* knockdown reduces expression of immune checkpoint receptors on CD8 + T cells and ligands on cancer cells. A) Expression of PD-1, CTLA-4 and LAG-3 in CD8 + T cells following 72 h of direct co-culture with MDA-MB-468 breast cancer cells. B) Expression of immune checkpoint receptors on CD8 + T cells following 72 h of indirect co-culture with MDA-MB-468 breast cancer cells. C) Expression of *PD-L1* and *CD86* in MDA-MB-468 cells following 72 h of indirect co-culture. Dot plots represent mean fold-change relative to siCTRL with standard error of mean (± SEM). Combined data from multiple independent experiments, each performed with one PBL donor (biological replicate), are shown. Statistical analysis performed using paired Student's t-test. *** *p* ≤ 0.001

Supplementary Material 6

## Data Availability

No datasets were generated or analysed during the current study.

## Abbreviations

7-AAD: 7-Aminoactinomycin D
Akt: Ak strain transforming
ATCC: American Tissue Culture Collection
BRCA: Breast cancer
CREB: CAMP-response element binding protein
CTA: Cancer testis antigen
CTL: Cytotoxic T lymphocytes
CTLA-4: Cytotoxic T-lymphocyte associated protein 4
CXCL1: Chemokine (C-X-C motif) ligand 1
CXCR2: CXC motif chemokine receptor 2
DEP: Differentially expressed protein
ELISPot: Enzyme-linked immunosorbent spot
EPIC: Estimating the Proportions of Immune and Cancer cells
FBS: Fetal bovine serum
FDA: Food and drug administration
Gal-9: Galectin 9
GM-CSF: Granulocyte-macrophage colony-stimulating factor
GO: Gene ontology
GSK-3B: Glycogen Synthase Kinase 3 Beta
HLA: Human leukocyte antigen
IFN-γ: Interferon gamma
IL: Interleukin
IPA: Ingenuity Pathway analysis
LAG-3: Lymphocyte activation gene 3 protein
LDHA: Lactate dehydrogenase A
LDHB: Lactate Dehydrogenase B
LDHC: Lactate Dehydrogenase C
LUMA: Luminal A
MAGE-C2: Melanoma-associated antigen C2
MCP-1: Monocyte Chemoattractant Protein-1
MDSC: Myeloid derived suppressor cell
MFI: Mean fluorescence intensity
NK: Natural Killer
OS: Overall survival
PBK: PDZ Binding Kinase
PBL: Peripheral blood lymphocytes
PBMC: Peripheral blood mononuclear cells
PCR: Polymerase chain reaction
PD-1: Programmed cell death protein 1
PD-L1: Programmed death ligand 1
PFS: Progression free survival
PI3K: Phosphoinositide 3-kinase
PPI: Protein–protein interaction
PRAME: Preferentially expressed antigen in melanoma
PVR: Poliovirus receptor
RNA: Ribonucleic acid
RPLPO: Ribosomal protein lateral stalk subunit P0
RPMI: Roswell Park Memorial Institute 1640
SFU: Spot forming units
siCTRL or siLDHC: Small interfering CTRL or LDHC RNA
Sp1: Specificity protein 1
SSX2: Synovial sarcoma X breakpoint 2
STAT: Signal Transducer and Activator of Transcription
T:E: Target:Effector ratio
TCGA: The Cancer Genome Atlas
TIDE: Tumor Immune Dysfunction and Exclusion
TIGIT: T cell immunoreceptor with Ig and ITIM domains
TIM3: T cell immunoglobulin mucin-3
TIMER: Tumor IMmune Estimation Resource
TPM: Transcripts per million
Treg: T regulatory cells
VISTA: V-domain immunoglobulin suppressor of T cell activation
