# Supplementary figures and images for: Immunomodulatory effects of tumor Lactate Dehydrogenase C (LDHC) in breast cancer
Source: Cell Commun Signal. 2025 Mar 19;23:145. doi: 10.1186/s12964-025-02139-6 (PMC11924725; doi:10.1186/s12964-025-02139-6)

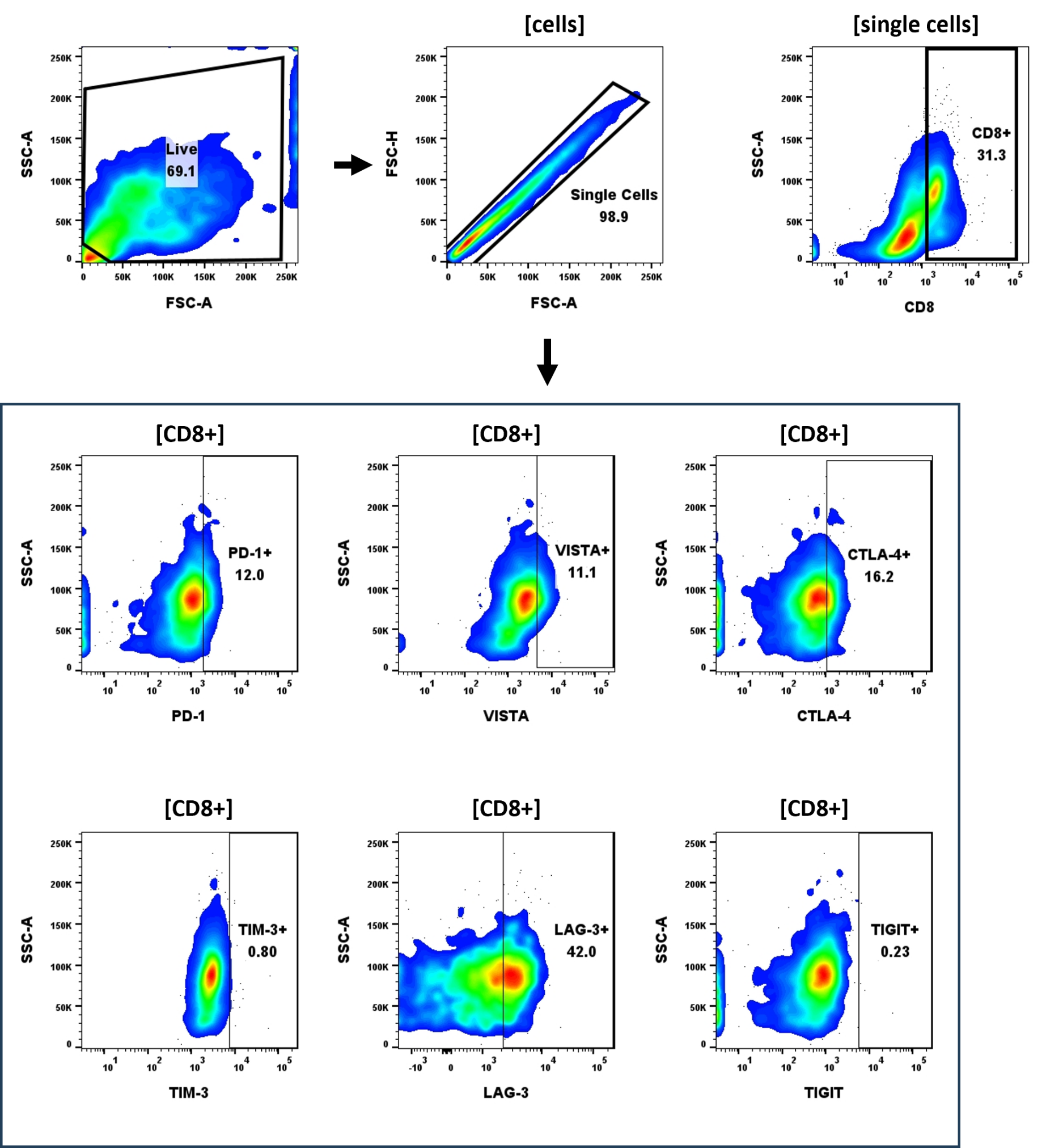

Supplement: Supplementary file 2 — Supplementary Material 2: Figure S1. Expression analysis of immune checkpoint receptors on CD8 + T cells. Flow cytometry plots depicting CD8 + T cell and immune checkpoint receptor gating strategies. Representative plots of one donor [file 12964_2025_2139_MOESM2_ESM.tiff]

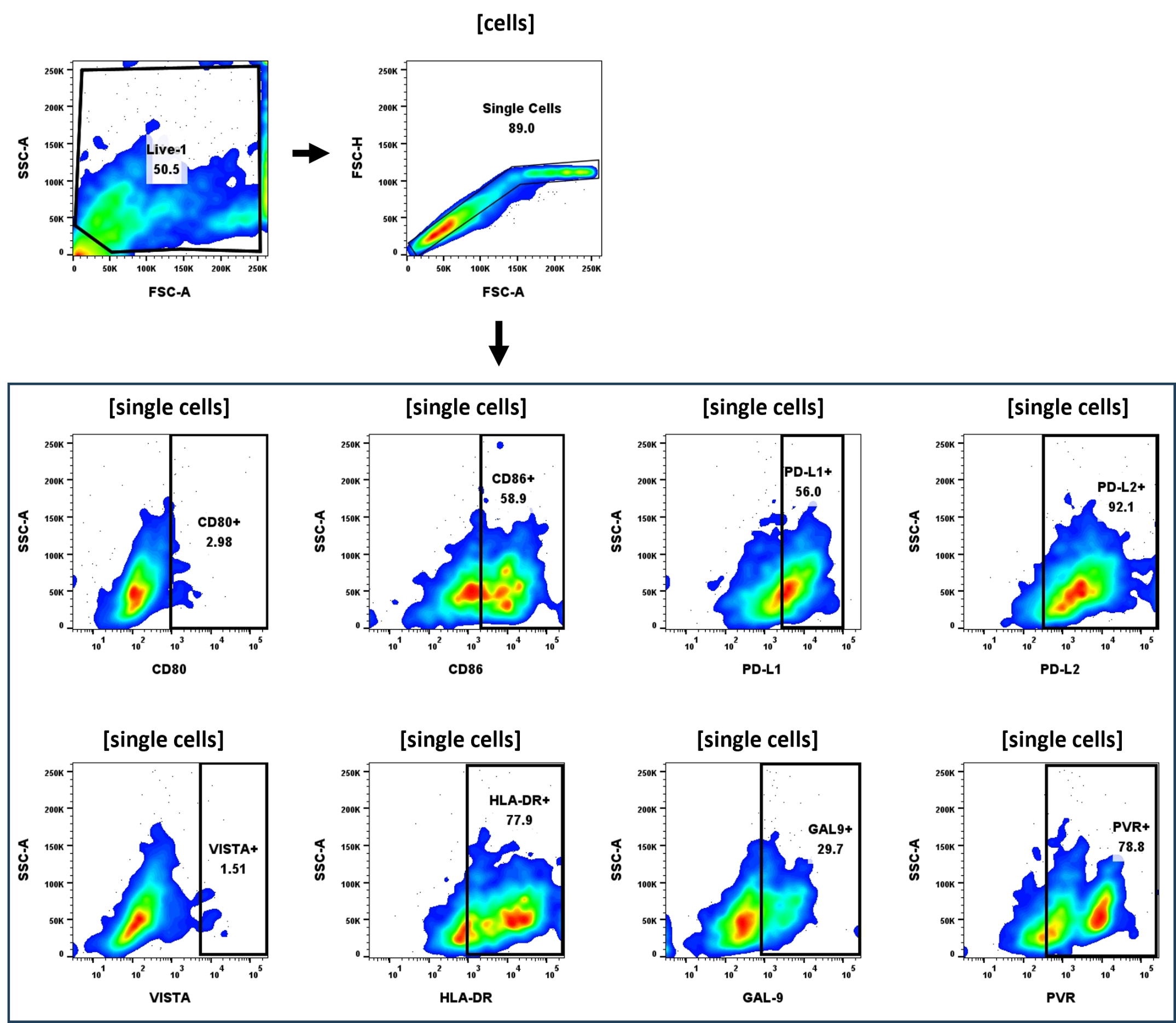

Supplement: Supplementary file 3 — Supplementary Material 3: Figure S2. Expression analysis of immune checkpoint ligands on tumor cells. Flow cytometry plots depicting immune checkpoint ligand gating strategies. Representative plots of indirect co-culture of cancer cells with peripheral blood lymphocytes from one donor [file 12964_2025_2139_MOESM3_ESM.tiff]

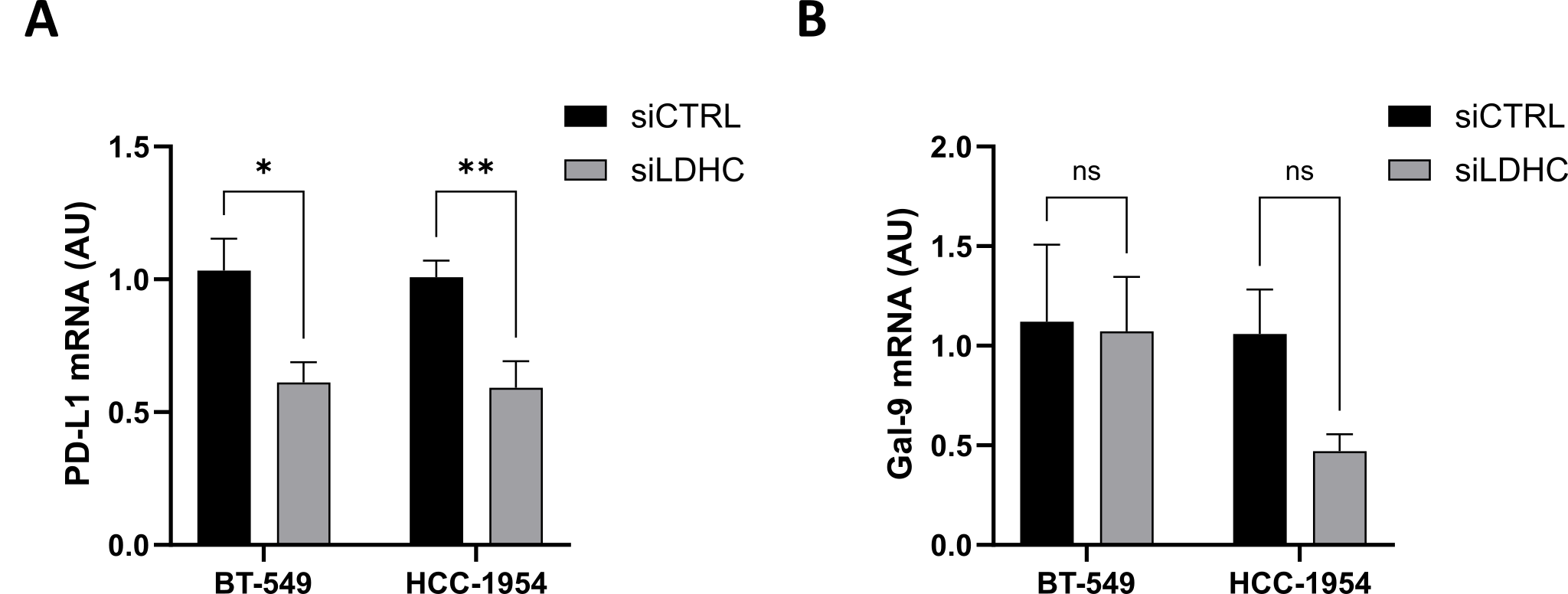

Supplement: Supplementary file 4 — Supplementary Material 4: Figure S3. LDHC knockdown reduces expression of immune checkpoint ligands on BT-549 and HCC-1954 breast cancer cells. A) PD-L1 and B) Gal-9 expression in BT-549 and HCC-1954 cells as measured by real time qRT-PCR. RNA expression data was normalized to RPLPO expression and plotted as mean fold-change relative to siCTRL. Combined data from a minimum of 3 independent experiments. Bar charts represent mean fold-change relative to siCTRL with standard error of mean (± SEM). Statistical analysis performed using paired Student's t-test. * p ≤ 0.05, ** p ≤ 0.01 [file 12964_2025_2139_MOESM4_ESM.tiff]

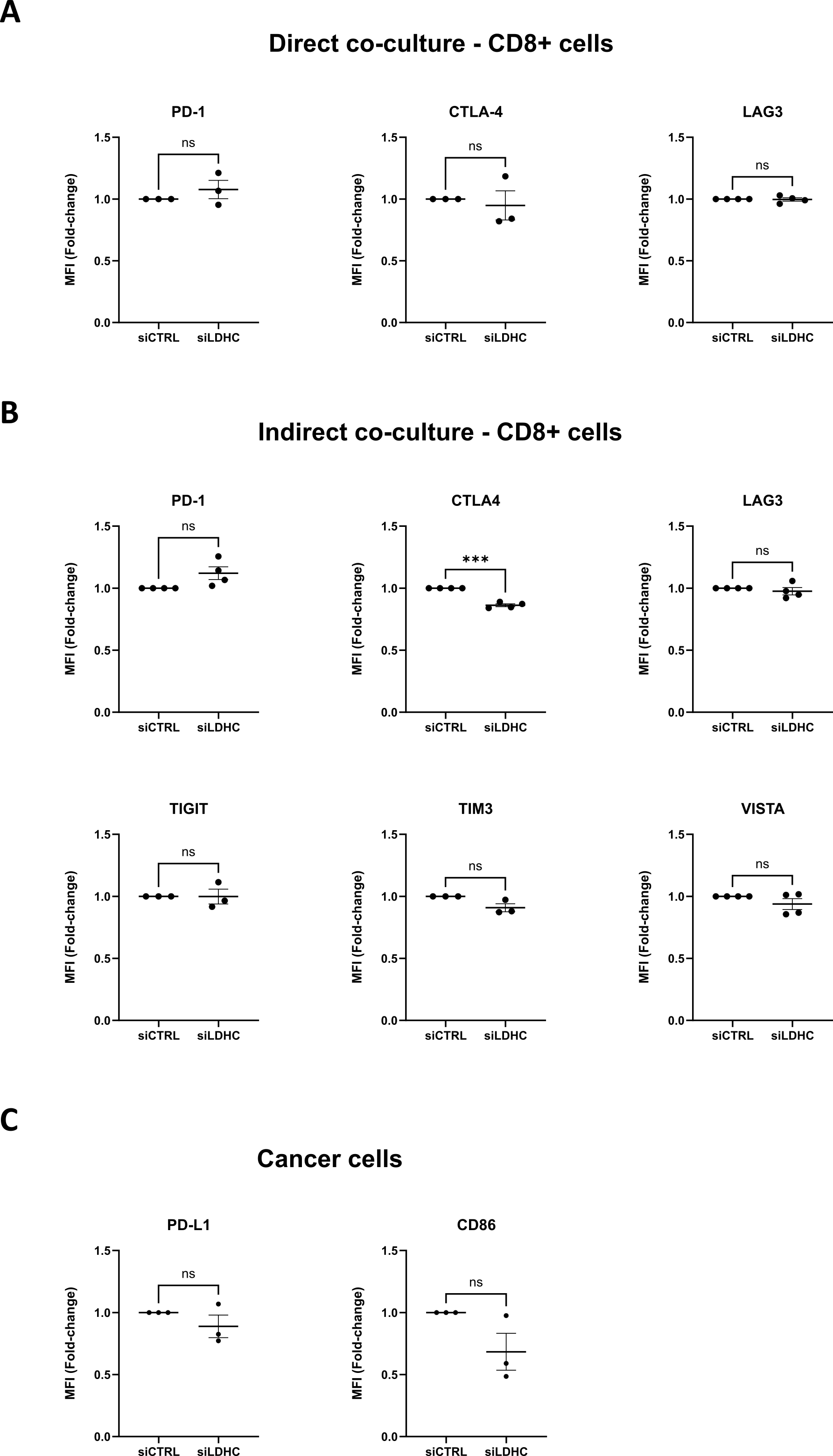

Supplement: Supplementary file 5 — Supplementary Material 5: Figure S4. LDHC knockdown reduces expression of immune checkpoint receptors on CD8 + T cells and ligands on cancer cells. A) Expression of PD-1, CTLA-4 and LAG-3 in CD8 + T cells following 72 h of direct co-culture with MDA-MB-468 breast cancer cells. B) Expression of immune checkpoint receptors on CD8 + T cells following 72 h of indirect co-culture with MDA-MB-468 breast cancer cells. C) Expression of PD-L1 and CD86 in MDA-MB-468 cells following 72 h of indirect co-culture. Dot plots represent mean fold-change relative to siCTRL with standard error of mean (± SEM). Combined data from multiple independent experiments, each performed with one PBL donor (biological replicate), are shown. Statistical analysis performed using paired Student's t-test. *** p ≤ 0.001 [file 12964_2025_2139_MOESM5_ESM.tiff]
