# Supplementary material for: Immunomodulatory effects of tumor Lactate Dehydrogenase C (LDHC) in breast cancer
Source: Cell Commun Signal. 2025 Mar 19;23:145. doi: 10.1186/s12964-025-02139-6 (PMC11924725; doi:10.1186/s12964-025-02139-6)
Supplement: Supplementary file 6 — Supplementary Material 6 [file 12964_2025_2139_MOESM6_ESM.pdf]

SUPPORTING MATERIAL: original blots for Figure 2B

MDA-MB-468

LDHC WB (~36kDa)   β-Actin WB (~45 kDa)

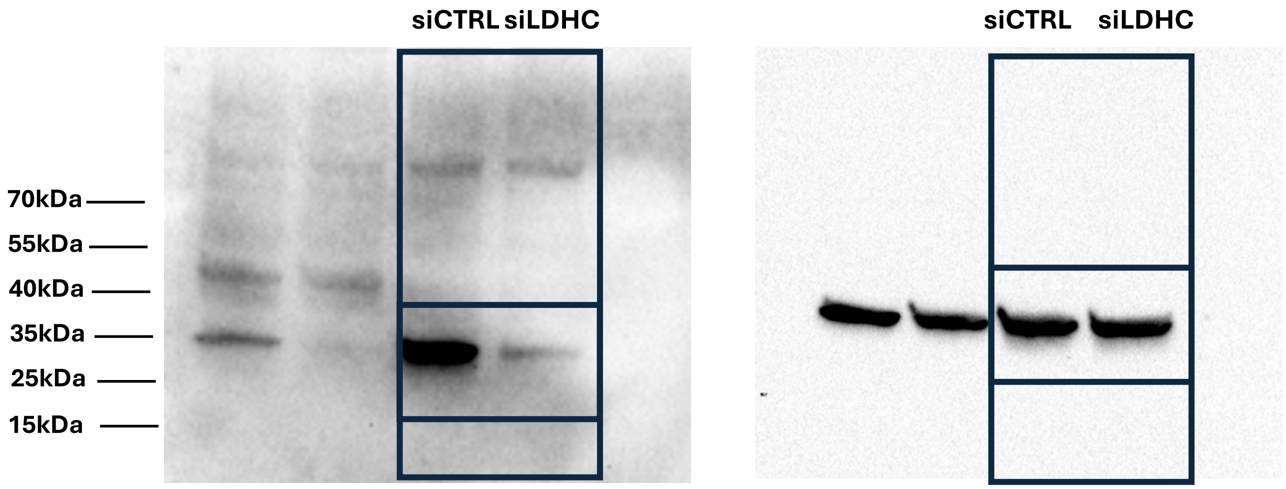

BT-549

LDHC WB (~36kDa)   β-Actin WB (~45 kDa)

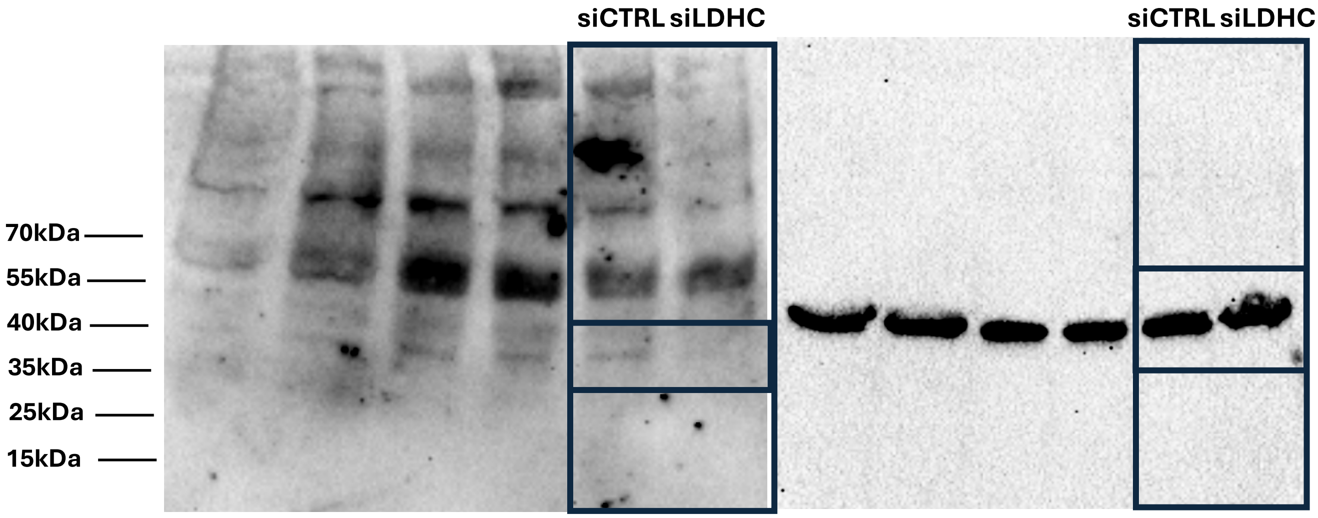

HCC-1954

LDHC WB (~36kDa)   β-Actin WB (~45 kDa)

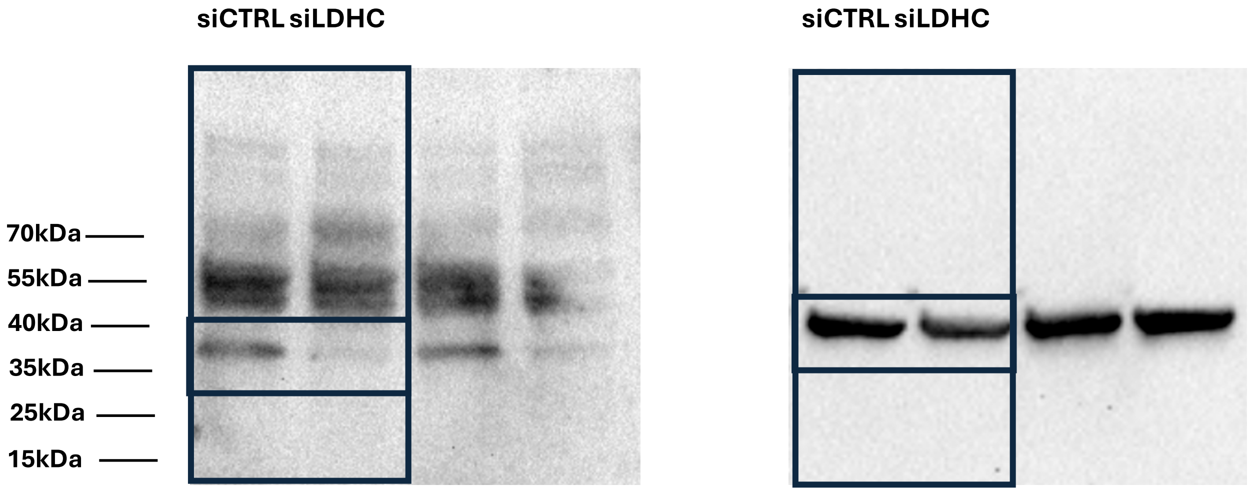

SUPPORTING MATERIAL: original blots for Figure 3C

MDA-MB-468

PD-L1 WB (40-50kDa)

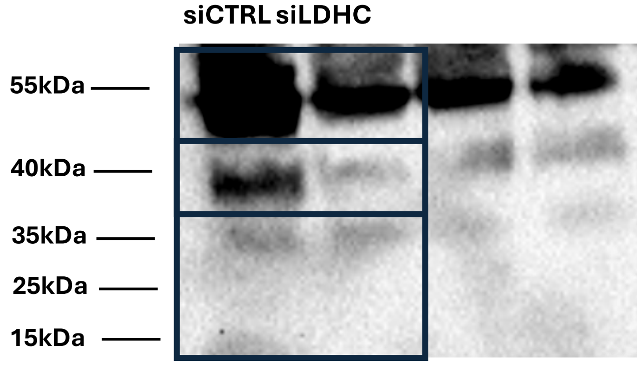

β-Actin WB (~45 kDa)

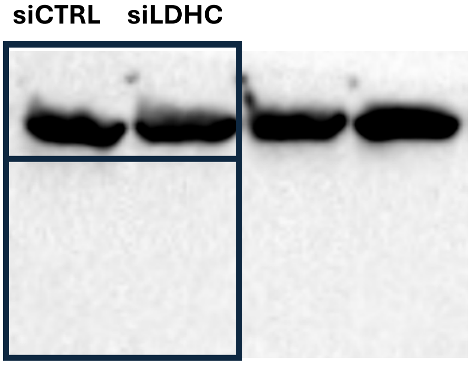

These images show the bottom half of the blot as it was cut in half to be probed with different antibodies for various experiments.
